# Pore Evolution in Cell Walls of Food Tissue during Microwave-Assisted Drying: An In-Depth Investigation

**DOI:** 10.3390/foods12132497

**Published:** 2023-06-27

**Authors:** Mohammad U. H. Joardder, Azharul Karim

**Affiliations:** 1 Department of Mechanical Engineering, Rajshahi University of Engineering and Technology, Rajshahi 6204, Bangladesh; 2 Faculty of Engineering and Science, Queensland University of Technology, Brisbane, QLD 4001, Australia

**Keywords:** microwave heating, temperature distribution, microstructure, nano–micro-pores, drying kinetics

## Abstract

Microwave (MW) heating is a unique approach that, unlike conduction- and convection-based heating, can provide volumetric heating. Complex microstructural changes in food materials occur because of simultaneous heat and mass transfer during drying, significantly affecting food structure and quality. Food properties, drying methods, and other drying parameters all have an impact on the microstructure of food samples, which in turn affects drying kinetics and food quality. However, no study has been undertaken to investigate the development of nano–micro-pores (NM-pores) on the cell walls and their relationship with the moisture migration mechanism. This study presents a novel investigation of the microstructural changes in food during microwave drying, with a focus on the formation of nano–micro-pores (NM-pores) on cell walls and their impact on moisture transport kinetics. The utilized hot air was maintained at a temperature of 70 °C, whereas microwave (MW) power levels of 100 W, 200 W, 300 W, and 400 W were used in microwave drying. The findings of the study indicate that the development of NM-pores occurs only during intermittent microwave drying (IMCD), while the cell wall of the food samples tends to burn or collapse in continuous microwave drying (CMD) due to the high heat generated. Additionally, no NM-pores were observed in the cell wall during convective drying. During IMCD with microwave power ranging from 100 W to 400 W, a range of pore sizes from 0.1 μm to 8.5 μm were observed. Due to the formation of NM-pores and collapses, MW drying takes around 10–20 times less time than convective drying to remove the same quantity of moisture. The effective moisture diffusivity values were found to be the highest in CMD at 4.70 × 10^−07^ m^2^/s and the lowest in CD at 2.43 × 10^−09^ m^2^/s. IMCD showed a moderate diffusivity of 2.45 × 10^−08^ m^2^/s. This study investigates the formation of NM-pores on cell walls during microwave drying and their impact on moisture transport kinetics and establishes correlations between microstructure modifications and moisture migration pathways.

## 1. Introduction

Most plant-based food materials are hygroscopic porous amorphous in nature with complex physical characteristics. The simultaneous heat and mass transfer process, which significantly changes the microstructure and quality of food, takes place during different types of food processing and preservation methods, such as drying, heating, cooking, baking, and frying. The physical structure and chemical composition of food material have an impact on the mechanisms that govern the mass and heat transfer. In order to identify the optimal drying conditions, researchers have conducted several studies exploring the correlation between microstructural changes and heat and mass transport in food products [1,2,3].

The microstructural changes and pore characteristics of food materials can vary depending on factors such as food properties, heating modes, and operating conditions [4,5]. The microstructural change in food is influenced by various heating conditions, including the temperature, relative humidity, and air velocity, as well as specific food properties, such as the composition, initial microstructure, and viscoelastic properties [6].

The issue of the quality of dried food has received considerable attention [7,8]. Crust formation, a longer drying time, and the poor quality of food are the main shortcomings of convective drying. The MW is not only energy efficient but it also significantly reduces processing time due to its volumetric heating capability [9]. Dipole water molecules rotate in the presence of the electromagnetic field during MW heating and the friction among the rotating water molecules causes heat generation. Eventually, the generated volumetric heat leads to the heating of water inside the cells and facilitates the process of evaporation. Nonetheless, the continuous supply of microwave power can lead to quality deterioration as it can cause an uneven distribution of temperature and moisture within the food material [10]. To address this issue, one approach to minimize the problem is to use an intermittent application of microwaves alongside other heating sources. Intermittent heating can be achieved by adjusting factors such as the temperature, air velocity, humidity, and operating pressure. Additionally, combining different drying modes can also help in reducing the negative effects of a continuous microwave power supply [11]. It is also attained by time-varying the application of a heat source.

Numerous studies on intermittent heating have been conducted, where the intermittency was accomplished by varying the heated air conditions. In recent years, the convenient use of microwave [12,13,14], radiofrequency [15,16], and ultrasound [17] methods has allowed researchers to use secondary energy sources intermittently. Intermittency in the energy input significantly influences the energy efficiency and product quality [14]. A large number of studies are available that discuss the temperature distribution in food samples during MW heating, but a complete insight of the mechanism of pores’ development on cell walls still eludes the researchers [18,19]. 

An outstanding challenge in these transport phenomena during the microwave heating of plant-based food cells is to relate the cell wall structure to the pore evolution mechanisms that affect values of diffusivity and instinct permeability. Pore formation during drying depends on both drying conditions and food sample properties [20,21,22,23]. The food cell wall, a flexible layer between 0.1 and 1 μm thick, is the boundary that separates cells with diameters in the range of 50–500 μm from the intercellular spaces (210–235 μm) [24,25] and can be considered a network of micro-fibers and nano-pores. The plant cell walls are comprised of water (about 60–75% by mass), cellulose micro-fibrils, hemicelluloses, cellulose, proteins, and pectin polysaccharides [26]. The cell walls can be treated as fiber-reinforced composite materials that show high elasticity in response to tension. As pectin forms a gel under specific conditions, cell wall micro-fibrils are kept apart and nano-scale cell wall pores are retained [27,28]. 

In general, plant-based tissue features extensive shrinkage and microstructural changes, such as a ‘softening’ or loss of ‘firmness’, during heating of any kind [29]. Due to the prolonged thermal exposure of the food cells, a significant amount of collapse can also take place. Utilizing microwaves increases vapor pressure differences between the interior and surface of the sample, developing a higher driving force for moisture flow. It can occasionally cause the sample to puff, while collapses and burning also potentially take place due to overheating. 

Rheological properties of the food matrix and heating conditions are the main factors that contribute to the cell wall morphological changes [30]. Apart from this, cell wall characteristics, such as the thickness, mechanical strength, cell geometry and dimension, and amount of bound water in the cell wall significantly affect the structural-changing phenomena. Therefore, it is difficult to predict which of the possible phenomena dominates in a sample [31]. Taking the pressure distribution and rheological properties of the cell wall into consideration, it can be claimed that the exerted pressure causes puffing if it is below the rupture energy of the cell wall. If the cell wall is sufficiently wet and at the rubbery phase, vapor pressure results in micro-pores’ evolution. However, if the cell wall is dried out sufficiently to attain a glassy phase and encounters a high vapor pressure, the cell wall may collapse permanently [32]. 

The microstructure and air content of the samples affect the dielectric properties as these determine the amount of water in the cells in food materials [33]. The fragile type of cell wall cannot sustain its rigidity under high vapor pressure. While the vapor is still migrating through the cell wall, the puffing of the cell also takes place. When the cell wall is sufficiently strong, highly pressurized vapor enlarges cell wall pores from a nano-to-micro scale. However, there is still minimal knowledge about details of pore enlargement during MW heating. Moreover, internal volumetric MW heating drives moisture from the intracellular spaces as well as cell walls. The bound water residing in the cell wall is also excited by the presence of microwaves, and many nano–micro-pores are developed on the cell walls. This type of phenomena is not found in the case of hot air convective heating.

The heating of food materials develops stresses owing to simultaneous thermal and moisture gradients. These stresses eventually cause cell deformation, which may cause cell ruptures during heating. The intensity of stress required for cell wall rupture highly depends on the mechanical properties of the plant cell wall as well as the modes of heating.

The existing literature lacks comprehensive research on the microstructural changes that occur in food during microwave drying, particularly regarding the formation of nano–micro-pores (NM-pores) on cell walls and their influence on moisture transport kinetics [23,24]. Although previous studies have examined the impact of microwave drying on food quality and drying kinetics, little attention has been given to the evaluation of microstructural changes and the development of NM-pores [25,26]. Therefore, this study aims to address this research gap by conducting an extensive investigation into the microstructural changes in food during microwave drying, with a focus on the formation and evolution of NM-pores. Therefore, an insightful understanding of the MN-pores’ evolution on the cell wall is essential to uncover the complex interactions between expanding cell wall pores and heat and water transport during MW heating. This study deals with the following aspects: (i) The formation of nano- and micro-pores (NM-pores) on the cell wall during microwave (MW) heating and their relationship with water transportation kinetics. This has not been previously studied in the context of MW heating; (ii) The challenge of relating the cell wall structure to pore evolution mechanisms that affect diffusivity and permeability values during the microwave heating of plant-based food cells. The findings of this study will contribute to the optimization of microwave-assisted drying processes and establish correlations between microstructure modifications and moisture migration pathways, leading to an enhanced food quality and improved drying efficiency.

## 2. Materials and Methods

### 2.1. Sample Preparation

After being brought from the local market of Brisbane, Australia, Granny Smith apples were stored in a refrigerator at 4 °C before being used for the drying experiments. Samples for heating experiments were discs of apple tissue with the dimension of 40 mm in diameter and 5 mm thickness. 

### 2.2. Experimental Apparatus

**Figure 1:**
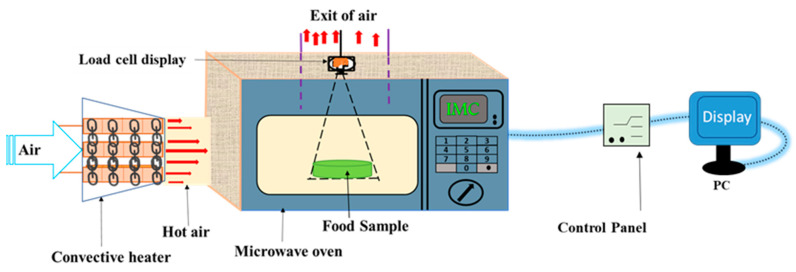
Schematic diagram of the IMC drying experimental setup.

The IMC drying system, depicted in Figure 1, comprises a convective heater and a modified inverter microwave oven (NN-SD691S, Panasonic, Osaka, Japan). The microwave oven can provide 10 precise power levels, reaching a maximum of 1100 W at a frequency of 2.45 GHz. By regulating the magnetron output, the microwave power level was controlled. 

**Figure 2:**
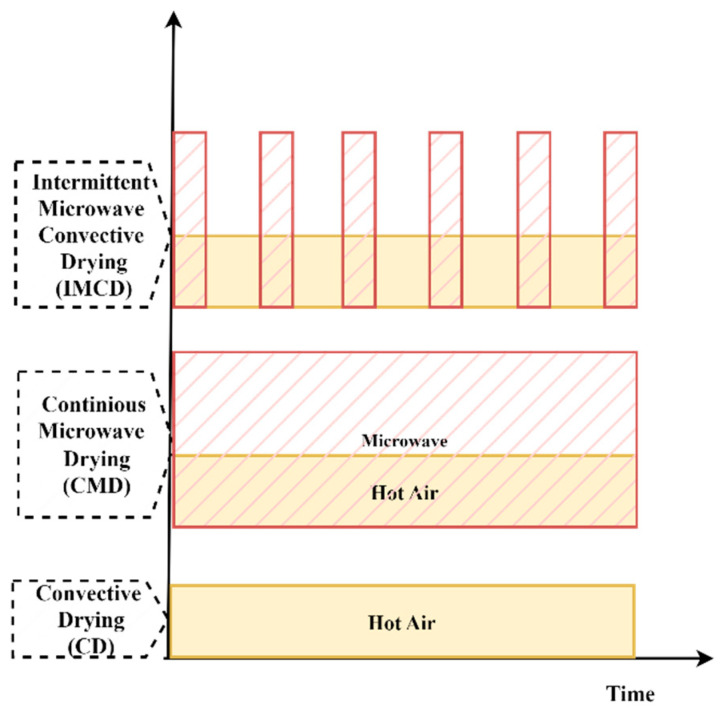
Hot air and microwave application in CD, CWD, and IMCD.

To generate convective hot air for the drying process, an electric heater was installed on a specific side of the microwave chamber. The experimental investigations were carried out under controlled conditions, maintaining a stable temperature of 70 °C. Furthermore, the inlet hot air velocity was carefully regulated to maintain a consistent flow rate of around 1 m/s throughout the experimental procedure. This arrangement ensured the simultaneous application of convective heating and microwave heating, enabling the exploration of the combined effects of both heating methods on the drying process. Three drying approaches, namely convective drying (CD), continuous microwave drying (CMD), and intermittent microwave convective drying (IMCD), have been investigated in this study, as shown in Figure 2.

Apple slices were placed in the center of the turn table of the microwave, where MW energy is distributed evenly. The sample was heated intermittently in a microwave for 60 s, followed by 120 s of convection in a convection drier. 

The temperature distribution on the surface of the samples was assessed using a FLIR i7 thermal imaging camera. It is important to note that accurate temperature measurement with thermal imaging cameras relies on appropriate emissivity values. In the case of apples, the emissivity value was determined to range between 0.94 and 0.97 [34], and the camera was set accordingly before taking images. 

### 2.3. Moisture Content Determination

Moisture content can be expressed as the quantitative amount of water present in a food sample on a wet or dry basis. Moisture content on a dry basis is defined as the mass of water that exists per unit mass of dry food materials. Using the following equation, moisture content on a dry basis has been determined. Using a load cell, real-time mass measurements were acquired to calculate the moisture content of the samples.

$$\mathrm{Moisture}\;\mathrm{content}\;(\mathrm{db})=\frac{weight_{wet}-weight_{dry}}{weight_{dry}}$$

### 2.4. Microscopic Observation and Image Processing

The Scanning Electron Microscope (SEM) model Quanta 200 (FEI company, Hillsboro, OR 97124-5793 USA), manufactured in Oregon, USA, was used to examine the microstructure of the dried apple slices. The SEM offers a range of magnification from 40× to 1000×, allowing for detailed observation of the sample at different scales. The SEM operates at an operating voltage of 20 kV, which optimizes the electron beam’s energy for effective sample analysis. By utilizing SEM images of dried apple slices, valuable insights of internal characteristics and properties, such as cell diameters, porosity, and pore distributions, can be analyzed.

**Figure 3:**
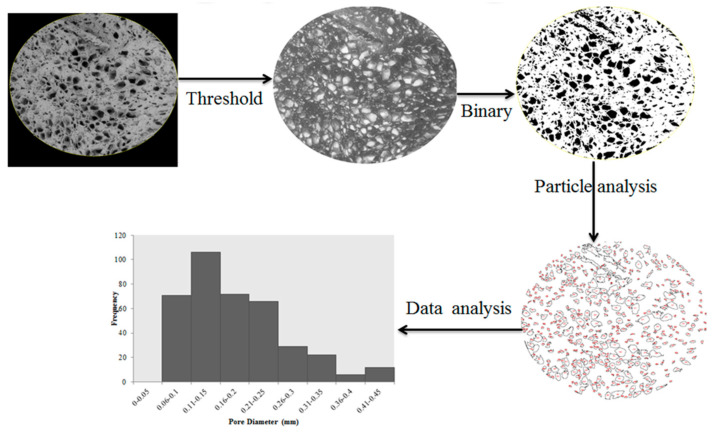
Determination of pore distribution using Image J software.

The quantitative analysis of the microstructure and pore distribution on the surface was conducted using Image J software (version 1.47v), which enables the measurement of pores’ area or size for accurate determination. The steps involved in pore distribution measurement are set out in Figure 3. Image processing using Image J typically involves several steps, including thresholding, binary image conversion, particle analysis, and data analysis. Once the threshold is applied, the image is converted into a binary image. The determination of pore sizes is conducted using particle analysis, which involves identifying and measuring the dimension of individual pores in the image. After particle analysis, the obtained data were further analyzed using a statistical method to find out the pore distribution.

## 3. Result and Discussion

### 3.1. Nano–Micro-Pore Formation

**Figure 4:**
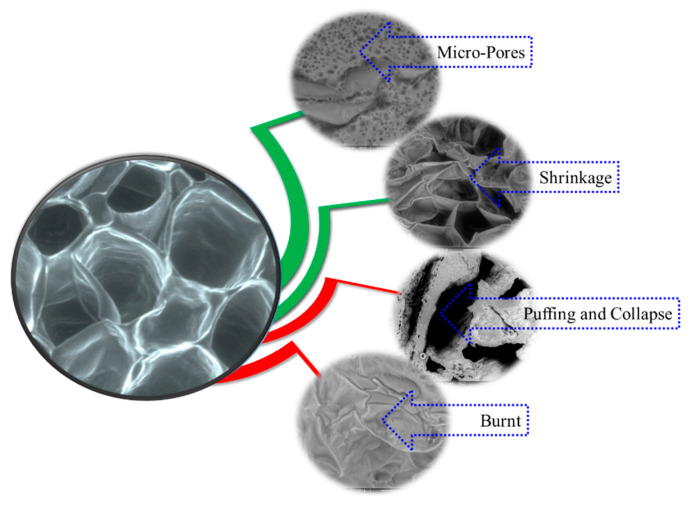
Morphological changes that take place during microwave heating.

**Figure 5:**
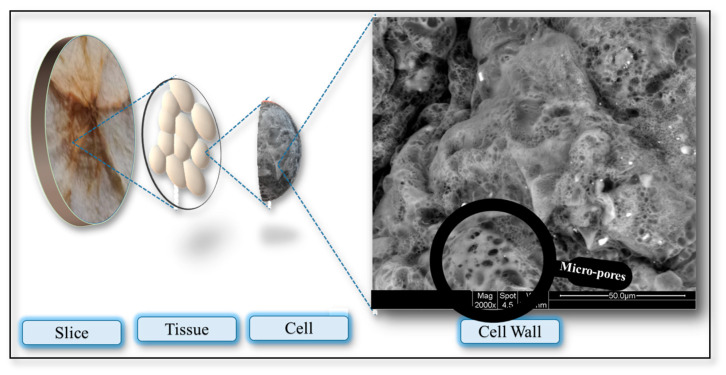
Micro-pore development on cell wall during microwave drying.

**Figure 6:**
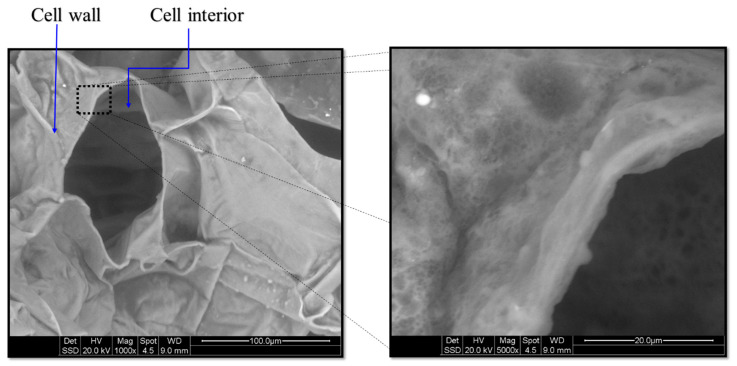
Scanning electron micrograph of convective drying of apple tissue at (1000–5000×).

Figure 4, Figure 5 and Figure 6 depict the microstructural changes during convective drying, CMD drying, and IMCD. Five distinct phenomena, namely, burning, collapse, shrinkage, puffing, and micro-pore formation on the cell wall, can be observed in microwave dried food materials, as shown in Figure 4. The MW power level, duration of MW application, and instantaneous moisture content as well as food types determine which types of phenomena take place during MW drying [35]. When the food materials are exposed to microwaves, local overheating takes place, as the warmer areas are susceptible to absorbing further energy [36]. Burning and puffing take place at the region of hot spots, as the heat absorption capacity of a hot portion, with an equivalent moisture content, is higher compared to its cold counterpart [37]. Similarly, due to the higher temperature inside the sample, rapid phase change happens eventually and puffing takes place. 

Internal evaporation also takes places during MW heating, which increases the partial pressure, resulting in a significant increase in the water migration rate [38,39], which causes structural changes [40].

When intermittency in microwave applications is used in conjunction with other heating methods, the sample frequently develops a consistent porosity on the cell walls as a result of the uniform distribution–redistribution of moisture and temperature [6]. 

CMD heating overheats some zones while keeping other places within the sample unheated. However, as shown in Figure 5, regulated drying kinetics during IMCD enable water to migrate in such a way that results in the development of numerous microscopic pores, which are not present in convective air-dried samples. 

Figure 6 shows the microstructure of apple tissue that was heated in convective drying. It is clearly depicted that most of the cells collapsed after convective heating. It is also depicted in the figure that the food sample’s inside is more porous than its exposed surface and that some of its cells are still intact. However, no micro–nano-pores (MN-pores) developed on the cell wall during convective heating. In response to stresses developed due to the thermal and moisture gradient during convection heating, cell walls either collapse or shrink [41]. 

In the case of continuous microwave drying, water is evaporated from the liquid phase within the tissue prior to migrating to the surroundings. When the evaporation rate inside the tissue is higher than the migration rate from the tissue, the excess vapor pressure is exerted on the cell wall. The pressure pushes the cell walls and therefore the adjacent intercellular spaces are brought together, making the cell wall stronger to withstand excess vapor pressure and avoid brakeage of the cell walls. Eventually, the puffing effect takes place due to this high vapor pressure during continuous microwave heating. 

In the case of IMCD, the developed vapor pressure is relatively smaller than that developed during continuous MW heating. Moreover, cell walls have a longer time to manage internal vapor pressure. In these distinct circumstances, cell walls respond differently. Unlike the sudden pressure development during CMD, gradual vapor pressure development and uniform pressure development take place during IMCD. This smooth and uniform pressure distribution during the intermittent application of MW heating results in micro pores’ evolution from the existing nano-pore of the cell wall. In summary, the temperature redistribution, rubbery state of the cell wall, and relatively lower vapor pressure exerted during IMCD collectively contribute to the development of NM-pores on the cell wall.

#### 3.1.1. Temperature Evolution and MN-Pore Formation

**Figure 7:**
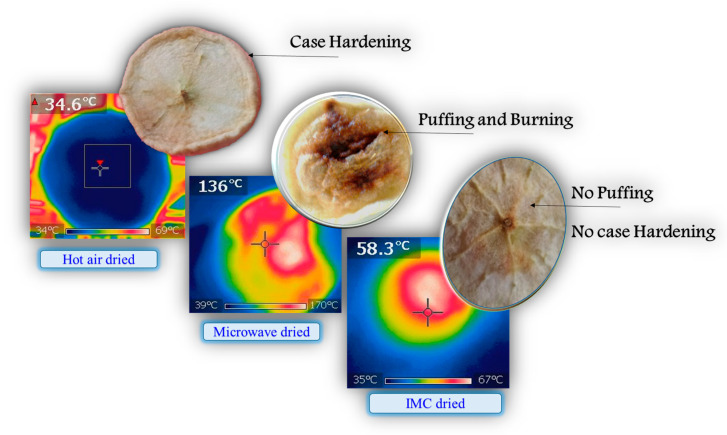
Temperature distribution during microwave and convective drying.

Figure 7 shows the temperature distribution in the food sample during convective, microwave, and intermittent microwave heating. The temperature distribution differs with the type of heating used on the food sample. Drying parameters in CD, CMD, and IMCD cause the temperature distribution. In convective heating, the center temperature is about 35 °C while the temperature in the edges is about 70 °C. Moreover, the continuous heating of exposed surface in convective heating causes case hardening, as shown in the figure. In the case of the continuous application of microwaves, hot spots with a significantly high temperature prevail, whereas rest of the sample maintains a comparatively lower temperature. 

The intensity of temperature has been found to have a significant effect on the pattern of cell wall shrinkage and collapse for plant-based foods [41,42]. During convective air heating, progressive internal thermal stress is developed, resulting in shrinkage and collapse [43,44]. Not all of the cells are broken at once; rather, cell breakage progresses in stages depending on the temperature distribution of the region that develops thermal stress in cells [6]. The thermal stress develops on the surface of the sample first and causes the breakage of those cells. Eventually, as shown in Figure 7, puffing and burning take place due to morphological changes during microwave heating. 

However, the lower spatial temperature gradient prevails in IMCD due to the tempering period that results in a more uniform temperature distribution. A non-uniform temperature distribution becomes relatively uniform once the redistribution of temperature takes place during the tempering phase of IMCD [45]. This alternate distribution and redistribution of the temperature during IMCD results in the development of micro-pores instead of causing puffing or burning.

The specific temperature distribution influences cellular level deformation differently as the extent of cell collapse or puffing varies with varying temperature distribution patterns. For instance, cell rupture starts from the outer surface of the food sample and progress gradually towards the core in convective heating [46].

**Figure 8:**
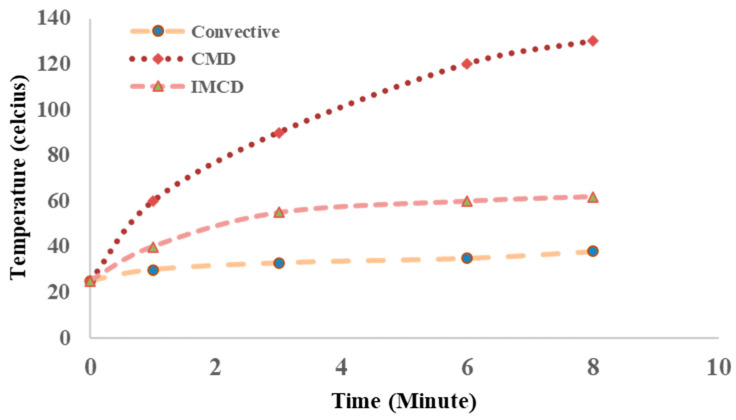
Average temperature of sample during CD, CMD, and IMCD in first 8 min of drying.

Figure 8 shows the average temperature of the sample rises in the first 8 min during CD, CMD, and IMCD. The different temperature levels reached during the drying processes have varying effects on the sample. In IMCD, where the temperature rises to 60 °C, moderate heat helps in moisture removal without causing significant damage to the sample. Convective drying at 40 °C provides a gentle drying environment, minimizing the risk of thermal degradation. However, CMD at 140 °C within 8 min exposes the sample to high temperatures, which can lead to undesirable effects, such as burning or the collapse of the sample’s structure.

#### 3.1.2. Relationship between MW Power Level and MN Pore Formation

**Figure 9:**
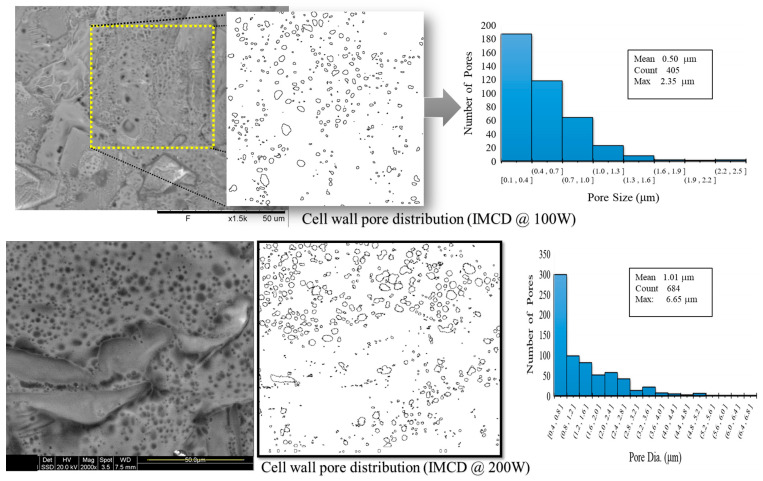
Cell wall pore distribution during IMCD at 100 W and 200 W.

**Figure 10:**
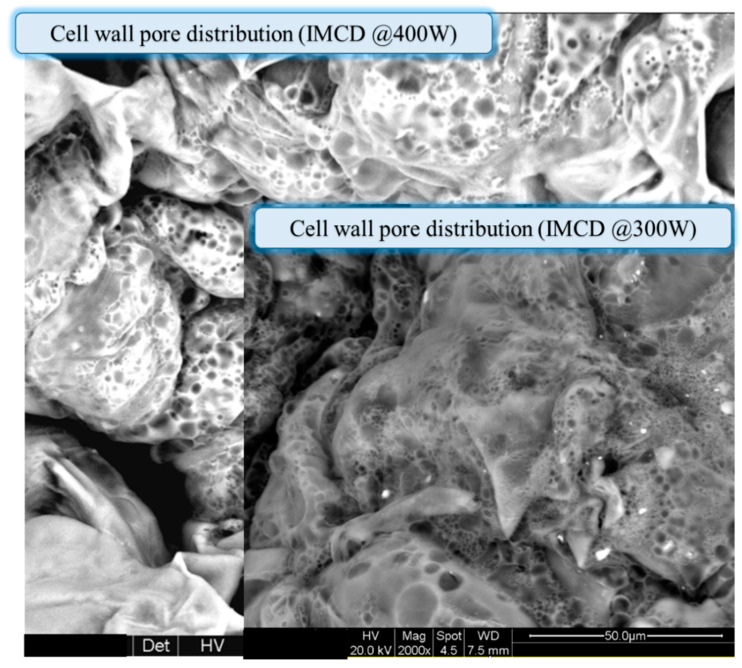
Effect of microwave power level on the micro pore formation.

The SEM pictures in Figure 9 and Figure 10 provide further insight into the effect of microwave power on pore formation in the dried apple samples. It can be observed that higher microwave power levels result in increased vapor pressure, leading to the formation of multiple tiny pores that eventually combine to form larger micro pores. For example, Figure 9 reveals the presence of pores with diameters ranging from 0.1 to 2.5 μm when the sample was dried at 100 W in IMCD. Figure 9 demonstrates the presence of pores with diameters ranging from 0.4–6.65 μm when the sample was dried at 200 W in IMCD.

Figure 10 reveals the presence of pores with diameters ranging from 1 to 8.5 μm when the sample was dried at 300–400 W. By adjusting the power appropriately, it is possible to control the size and number of pores, which can have significant implications for the quality and characteristics of the dried product. 

Taking the effect of MW power into consideration, the overheating and cell collapse of the dried samples during CMD can be effectively controlled by manipulating both the microwave power and intermittent application. The application of continuous microwaves is known to cause overheating and the collapse to the cell walls [47]. In contrast, IMCD offers opportunities for maintaining the texture and porosity of the final product, allowing for the optimization of desired characteristics. By carefully adjusting the microwave power and time of intermittent MW application, it becomes possible to achieve the desired porous cell wall and enhance the overall quality of the dried samples. 

### 3.2. Significance of MN-Pores’ Formation in Moisture Migration

Table 1 presents the effective moisture diffusivity, drying time, and specific energy consumption of apple drying in CD, CMD, and IMCD. The table shows that convective heating takes much longer to remove moisture compared to microwave heating. In convective drying, heat travels from the outside to the core of the apple, which requires overcoming the resistance posed by the plant tissue. This results in the outer part of the apple drying first, leading to case hardening. As a result, the overall ability of the apple to conduct heat and allow moisture to move decreases significantly [29].

CMD removes the same amount of moisture in 20 and 2.5 times less time than convective drying and IMCD, respectively. The liquid moisture inside the samples evaporates before it migrates out of the cell, creating a sizable pressure gradient that, depending on the cell’s surrounding environment, either causes the cell to puff up or collapse. Puffing affects the overall rate of water migration out of the cell [48]. More water molecules move because of increased pressure-driven flow in addition to the usual diffusion-driven flow. Chen et al. [37] refer to a similar observation in the high-power microwave drying of apples. However, the persistent MW heating leads to localized overheating, which burns cell walls and opens wider channels for water migration. This observation is in agreement with the findings of Izli et al. [49], which showed that IMCD takes a relatively shorter time than convective drying. 

It is hypothesized that IMCD enhances water diffusion in the presence of NM-pores, resulting in faster water migration compared to convective heating but slower water migration than continuous microwave heating due to intact cell walls. The movement of water during intermittent microwave heating is influenced by pressure gradients and cell wall properties, such as permeability and the presence of small pores. The permeability of the cell wall is affected by the micro-fiber structure and pore characteristics. Cell walls with small pores facilitate greater water movement from inside the cells to intercellular spaces, leading to higher permeability. This increased permeability allows water molecules to flow more quickly during intermittent microwave heating compared to convective heating.

In summary, the absence of NM-pores in the cell wall indicates that the moisture migration pathway may rely on different mechanisms during convective drying. Convective drying relies on heat transfer through the surrounding air, which results in a different moisture removal behavior compared to microwave-assisted drying methods. The formation of NM-pores during IMCD is a significant contributor to moisture transport kinetics. These pores play a crucial role in facilitating moisture migration within the food samples. The increase in microwave power leads to the growth of NM-pores in terms of both size and number. This phenomenon can be attributed to the distinct temperature distribution and moisture evaporation pattern during IMCD, whereas CMD results in the overheating and burning of the food samples. The excessive and continuous application of microwave power in CMD can cause damage to the cell wall structure, leading to undesirable quality and nutritional properties of the food. The absence of NM-pores in CMD suggests that the moisture transport mechanism may differ from that in IMCD.

## 4. Conclusions

During drying, complex microstructural changes occur due to concurrent heat and mass transfer, which significantly affect the drying kinetics and quality. The study findings reveal that NM-pores are exclusive to IMCD, as CMD leads to cell wall burning or collapse, while convective drying shows no NM-pore formation. The data show a direct correlation between microwave power and pore growth. IMCD effectively mitigates issues of overheating and burning while being significantly faster than convective heating. The formation of NM-pores on the cell walls during IMCD is a significant contributor to moisture transport kinetics. The number and size of pores increase with an increase in microwave power, which is influenced by the distinct temperature distribution and moisture evaporation pattern. These findings highlight the distinct effects of different drying methods on the microstructure of food samples. The presence of NM-pores in IMCD suggests that it may be a favorable drying method for preserving the quality and nutrients of the food, as the moisture transport kinetics are facilitated without causing significant damage to the cell wall. The successful application of the findings would offer uniform drying conditions and improved drying efficiency. Understanding the formation and behavior of NM-pores on cell walls during microwave drying could lead to the development of optimized drying protocols. Further research is required to fully understand the underlying mechanisms and optimize the MW drying processes to enhance the quality of dried food products.

## Figures and Tables

**Table 1:** Effective moisture diffusivity, drying time, and specific energy consumption for apple sample drying using CD, CWD, and IMCD.

Drying Condition	Drying Time (min)	Effective Diffusivity (m^2^/s)	SEC (kJ/kg)
Convective Drying	200	2.43 × 10^−09^	153,500.7
CMD	10	4.70 × 10^−07^	1666.67
IMCD	25	2.45 × 10^−08^	4444.44

## Data Availability

Data are contained within the article.
